# Beyond Biology: Impact of Center- and Country-specific Economic Factors on Outcomes After Hematopoietic Cell Transplantation

**DOI:** 10.1016/j.ebiom.2015.12.005

**Published:** 2015-12-12

**Authors:** Nandita Khera

**Affiliations:** College of Medicine, Mayo Clinic, 5777 E Mayo Blvd, Phoenix, AZ 85054, United States

**Keywords:** Hematopoietic cell transplantation

Ever since the first hematopoietic cell transplantation (HCT) was performed in 1960s, thousands of studies have elucidated the impact of patient and donor factors (sociodemographic, disease and transplant characteristics) on outcomes after the procedure. Fewer studies have looked at the role of center specific factors such as procedure volume, center experience or accreditation status in influencing the outcomes (Loberiza et al., 2005, Horowitz et al., 1992, Gratwohl et al., 2014). Macroeconomic factors such as gross national income per capita or health care expenditure per capita have been shown to impact the diffusion and utilization of HCT, because it is an expensive and resource intensive technology (Gratwohl et al., 2013, Gratwohl et al., 2002). However, the impact of these factors on outcomes hasn't been well studied especially in the context of individual patient-level and center-specific factors.

In this issue of EBioMedicine, Baldomero et al. present a retrospective population level analysis that examines the interplay of patient-, center- and country level factors on outcomes of allogeneic and autologous HCT using data from the European Society of Blood and Marrow Transplantation (EBMT) database (Baldomero et al., 2015). The authors use a large patient cohort with a long follow-up of 8 years from 404 HCT centers in 25 European countries and incorporate center- and country specific economic data into a detailed multi-level analysis. They describe the association of program accreditation and duration, patient volume, human development index, gross national income/capita, and health care expenditures/capita with clinical outcomes (overall survival (OS), non-relapse mortality (NRM) and relapse) after HCT while adjusting for patient related factors. They report accreditation, higher patient volumes and longer program duration as center properties associated with better overall outcomes. These favorable center characteristics are more common in affluent countries and may explain in part the better survival, decreased NRM and relapse risk after allogeneic HCT in countries with higher economic indices. However, the authors rightly note that this relationship cannot be determined as causal because of the nature of the study and analysis. The relationship between outcomes and center- and country-specific factors is less definitive in the case of autologous HCT.

The findings about the differential impact of macroeconomic factors on survival after autologous and allogeneic HCT are novel, but not surprising as these are two different types of procedures with different degree of medical complexity, risk and resource requirement. Even though the study is about HCT in Europe, it has wider implications. An important question it raises is ‘if one size fits all?’ for worldwide practice of HCT or for that matter, any expensive medical technology. Should the benchmarks for assessing the success of an expensive technology be different amongst different countries at diverse stages of socioeconomic development with different resource capacity (knowledge base, human resources and institutional infrastructure)? The authors suggest streamlining and consolidation of the transplant activity to help maintain the quality of care and strengthening the overall health infrastructure to provide optimal pre- and post-HCT care. This proposition and a long term vision for the growth and sustainability of a transplant program may be more important for the poorer countries than the richer countries to help optimize the best utilization of the scarce resources. This is because the spending on health care especially on high cost medical technology is more likely to strain the government as well as individual household budgets in poorer countries as opposed to affluent countries. The bigger question is if the poorer countries should spend their limited resources to improve outcomes of expensive procedures that benefit only a few or focus on providing basic necessities and preventive care for a larger population?

The authors highlight the role of professional medical societies worldwide in improving the quality of care and scaling up transplant systems through education and training that fit within the economic framework of individual countries across the globe. This will need to be supplemented with other measures such as international aid, advocacy efforts, partnerships and investment in research in the affluent countries to develop affordable technologies. Bidirectional knowledge transfer is important because the evolution of a technology developed in a richer country may follow a more cost-effective route when implemented in developing countries. This may provide an opportunity for the affluent countries to learn a less expensive way to do things as long as it doesn't impact short- and long-term outcomes (Ruiz-Delgado, 2012). Finally, the policy makers need to realize the importance of bridging the divide between macroeconomic policy and health policy to commit to building the necessary scientific and research capacity that can optimally address the medical needs of the country through careful resource allocation. We have crossed the one million mark for HCT across the world, now we need to strive to achieve a compromise between equity and efficiency.
